# Association between rs4673 and blood pressure response to acute saline infusion in Chinese population

**DOI:** 10.1097/MD.0000000000041463

**Published:** 2025-02-14

**Authors:** Hongyi Wang, Xue Wang, Yan Tian, Li Yang, Xiaoxia Han, Zhuo Wang, Xiaoyan Nie, Ningling Sun

**Affiliations:** a Department of Hypertension, People’s Hospital, Peking University, Beijing, China; b Department of Pharmacy Administration and Clinical Pharmacy School of Pharmaceutical Sciences, Peking University, Beijing, China; c Beijing E-Seq Medical Technology Co. Ltd., Beijing, China; d Yan’an Affiliated Hospital of Kunming Medical University, Yunnan, China; e Qingxu County People’s Hospital, Shanxi, China.

**Keywords:** blood pressure, CYBA protein, essential hypertension

## Abstract

This study investigated the relationship between rs4673 of the nicotinamide adenine dinucleotide phosphate oxidase p22PHOX gene and blood pressure (BP) response to acute salt loading in a Chinese population diagnosed with essential hypertension. An acute salt loading test was performed using 2 L of normal saline (NS) infused over 4 hours. BP and heart rate were recorded immediately after NS infusion and hourly for the next 3 hours. Data analysis was performed using the chi-squared test, Student *t* test, and multivariable regression. A total of 159 patients were analyzed, including 129 with the CC genotype, 29 with the CT genotype, and 1 with the TT genotype. Individuals carrying the T allele exhibited greater tolerance to BP increases caused by 2 L NS infusion. Specifically, the systolic blood pressure change for T carriers was 0.26 ± 9.72 mm Hg, compared to 6.82 ± 11.65 mm Hg for those with the CC genotype (*P* = .005). Diastolic blood pressure changes were −3.35 ± 7.52 mm Hg in T carriers versus 1.38 ± 7.62 mm Hg in CC genotype carriers (*P* = .003), and mean arterial pressure changes were −2.13 ± 7.85 mm Hg in T carriers compared to 3.19 ± 7.81 mm Hg in CC genotype carriers (*P* = .001). These significant differences persisted after adjusting for gender, age, smoking, drinking, and baseline BP. Interestingly, not all subjects experienced increased BP following NS loading; 86.82% in the CC group did, versus 66.67% of T allele carriers (*P* = .014). The findings suggest that individuals carrying the T allele are less likely to be salt-sensitive, as indicated by a diminished BP response to acute saline infusion. This contributes to the understanding of the genetic factors that influence salt sensitivity in essential hypertension.

## 1. Introduction

Hypertension is a multifaceted disease influenced by both genetic and environmental factors, which significantly elevates the risk of cardiovascular disease, the predominant cause of global disease burden. Among the environmental factors, dietary salt intake is a critical component implicated in the onset of hypertension.^[1]^ Nonetheless, individuals exhibit varied blood pressure (BP) responses to changes in their salt intake. The Genetic Epidemiology Network of Salt Sensitivity study revealed that 33% to 61% of Chinese subjects experienced BP increases with high-salt intake, while 33% to 62% showed decreases with low salt intake, and 23% to 31% exhibited no significant change or a reverse change.^[2]^

The pathogenesis of hypertension and salt sensitivity is intricate, involving endothelial dysfunction, which is closely linked to both conditions.^[3]^ Endothelial dysfunction denotes the impaired vasodilation capacity primarily due to diminished production or availability of vasodilators such as nitric oxide (NO). This dysfunction is often precipitated by excessive levels of reactive oxygen species (ROS) or other inflammatory agents, which promote plaque formation and smooth muscle cell proliferation.^[4]^ Moreover, elevated ROS levels pose a significant risk factor for various ailments, including atherosclerosis, diabetes, coronary artery disease, myocardial infarction, end-stage kidney disease, ischemic stroke, and cancer, among other diseases.^[5–10]^

Nicotinamide adenine dinucleotide phosphate (NADPH) oxidase (NOX) is a critical enzyme complex responsible for generating superoxide anions, hydrogen peroxide, and NO, constituting the primary sources of ROS.^[11]^ The NOX family, characterized by its cytoplasmic (p46PHOX and gp91PHOX) and membrane-bound (p67PHOX and p22PHOX) subunits, plays a pivotal role in ROS production. Within this complex, p22PHOX is essential for stabilizing gp91PHOX and facilitating superoxide generation, thereby directly influencing the assembly and activity of the NOX complex.^[11,12]^ The rs4673, also known as C242T, in the p22PHOX (Officially known as CYBA) gene, which encodes the p22phox protein, is a missense mutation characterized by a substitution of tyrosine at position 72 with histidine, leading to a loss of function in the p22phox binding domain.^[12,13]^ Population-based studies on coronary artery disease patients have indicated that carriers of the rs4673 allele exhibit significantly lower vascular NOX activity.^[14]^

Evidence from animal model experiments supports the association between excessive ROS levels and salt-sensitive hypertension. Studies involving Dahl salt-resistant (DRS) and Dahl salt-sensitive rats on high-salt diets demonstrated that Dahl salt-sensitive rats developed hypertension, characterized by decreased nitric oxide synthase expression and increased vascular NOX expression compared to normotensive DRS rats.^[15,16]^ Treatment with antioxidants or NOX inhibitors has been shown to mitigate salt-induced hypertension and vascular superoxide levels. Supplementation with NO also attenuated the rise in salt-sensitive BP in DRS rats responding to a high-salt diet.^[17]^

However, the potential association between the rs4673 polymorphism and salt sensitivity in the Chinese population remains underexplored. This study aims to fill this gap by identifying the rs4673 genotype through sequencing in patients with essential hypertension and analyzing its association with BP response to acute salt loading.

## 2. Materials and methods

### 2.1. Study population

This study included inpatients newly diagnosed with primary grade 1 hypertension, aged 18 to 60 years, who had not previously used antihypertensive medication and had provided informed consent. Patients with any of the following conditions were excluded (1) grade II and above hypertension; (2) myocardial infarction; (3) heart failure; (4) stroke; (5) aortic dissection; (6) atherosclerosis; (7) renal insufficiency; (8) metabolic syndrome; (9) helicobacter pylori infection; (10) diabetes; (11) tumor; (12) liver disease. This study was approved by the Ethics Committee of Xiangya Hospital of Central South University, China (R17014). Informed consent was obtained from all patients participating in the study.

### 2.2. Salt loading and measurements

Upon admission, patients adhered to a normal sodium diet. On the day following hospital admission, each participant was equipped with an ambulatory BP monitor for 24-hour mean BP and hear rate (HR) monitoring, including daytime (6:00 am–10:00 pm) and nocturnal (10:00 pm–6:00 am) periods. Furthermore, a 24-hour urine collection was performed to assess sodium and potassium levels. Baseline data also included age, gender, body mass index, and factors such as, obesity, smoking, and alcohol consumption. On the third day, after resting BP measurements at 7:50 am, all patients received an infusion of 2 L normal saline (NS) over 4 hours, starting at 8:00 am.^[18]^ BP and HR measurements were recorded immediately after the NS infusion concluded and then hourly for the subsequent 3 hours. At each time point, measurements were taken 3 times to calculate a mean value.

### 2.3. Genotype assay

Genomic DNA was extracted from oral mucosal epithelial cells using a disposable oral exfoliated cell sampling box (Taitong Gene Testing, Suzhou, China), following standard procedures. The G to A substitution, corresponding to the GRCh37.p13 chr 16 sequence, was identified by polymerase chain reaction using primers designed from the NCBI website. The cycling conditions were set to 90 °C for 30 seconds, 45 cycles at 50 °C for 5 seconds, and 72 °C for 5 seconds, followed by a denaturation step for 1.5 minutes at 90 °C and high-resolution melting curve analysis from 40 °C to 70 °C. The genotype success rate for this site reached 100%.

### 2.4. Statistics analysis

Data were analyzed using STATA version 15.1 and SPSS 16.0™. Two-sided hypothesis testing was performed with a *P*-value < .05 considered statistically significant. Categorical variables were described with occurrence numbers and percentages, and compared between genotype groups using the Chi-square test. Continuous variables were presented as mean ± standard deviation and analyzed using ANOVA for normally distributed data, or the Kruskal–Wallis test for non-normally distributed data. Linear multivariable regression analysis was utilized to adjust results for gender, age, smoking, drinking, and baseline BP. The Hardy–Weinberg equilibrium test was conducted using SHEsis software.^[19]^

## 3. Results

### 3.1. Participants’ genotype of rs4673

A total of 159 patients were enrolled in this study. The rs4673 genotype and allele frequencies of participants were presented in Table 1. The distribution was as follows: CC genotype in 129 individuals (81.13%), CT genotype in 29 individuals (18.24%), and TT genotype in 1 individual (0.63%). The frequency for C allele and T allele was 90.6% and 9.4%, respectively (*P*-value for Hardy–Weinberg equilibrium is .426), consistent with allele frequencies observed in the general East Asian population from the National Center for Biotechnology Information (NCBI).

**Table 1:** The rs4673 genotype and allele-frequency of participants.

Genotype	Genotype (n, frequency)	Allele	Allele-frequency	HWE *P*
CC	129 (0.811)	C	0.906	.426
CT	29 (0.182)	T	0.094	
TT	1 (0.007)	–	–	–

HWE = Hardy–Weinberg equilibrium.

### 3.2. Association between genotype and participants characteristics

As indicated in Tables 2 and 3, there were no significant differences between the 2 genotype groups for key variables including gender, age, body mass index, obesity, smoking status, alcohol consumption, 24-hour urinary sodium and potassium excretion, mean 24-hour (systolic blood pressure) SBP/diastolic blood pressure (DBP)/HR, mean daytime SBP/DBP/HR, mean nighttime SBP/DBP/HR, changes in HR from day to night, and the prevalence of non-dippers.

**Table 2:** The demographics of the studied population.

Variable (c.214T > C)	CC (n = 129)	CT + TT (n = 29 + 1)	*P*
Gender (male, n, %)	83 (64.34)	18 (60.00)	.912
Age (year)	44.42 ± 12.61	42.07 ± 11.34	.645
BMI (kg/m^2^)	25.53 ± 3.50	25.38 ± 2.63	.977
Smoking (n, %)	48 (37.21)	6 (20.00)	.200
Drinking (n, %)	52 (40.31)	8 (26.67)	.412

*Note:* Categorical variables are presented as the number of occurrences and percentages. Continuous variables are reported as mean ± standard deviation (SD). Categorical variables were compared using the Chi-square test, while continuous variables were analyzed using one-way ANOVA.

BMI = body mass index.

**Table 3:** The baseline characteristics of the studied population.

Variable (c.214T > C)	CC (n = 129)	CT + TT (n = 29 + 1)	*P*
24 h mean SBP	131.71 ± 12.04	132.4 ± 11.44	.956
24 h mean DBP	85.92 ± 10.23	86.20 ± 9.32	.991
Daytime mean SBP (mm Hg)	134.73 ± 12.05	135.53 ± 11.40	.947
Daytime mean DBP (mm Hg)	88.14 ± 10.07	88.63 ± 9.43	.970
Nighttime mean SBP (mm Hg)	123.27 ± 14.17	124.73 ± 11.97	.707*
Nighttime mean DBP (mm Hg)	79.40 ± 11.38	79.5 ± 9.86	.872
Non-dippers (n, %)	70 (54.26)	21 (70.00)	.304
24 h mean HR	75.24 ± 9.27	73.17 ± 6.80	.551
Daytime HR	78.57 ± 10.01	75.83 ± 7.65	.481
Nighttime HR	66.07 ± 9.11	65.00 ± 7.40	.857
Day–night change HR	-12.54 ± 5.94	-10.83 ± 5.48	.556
24 h UNa (mmol)	161.45 ± 79.37	164.08 ± 75.41	.968*
24 h UK (mmol)	40.65 ± 13.56	42.03 ± 16.97	.999*

Daytime is defined as 6:00 am to 10:00 pm; nighttime is defined as 10:00 pm to 6:00 am. Categorical variables were reported as the number of occurrences and percentages and were compared using the Chi-square test. Continuous variables were presented as mean ± standard deviation (SD) and compared using one-way ANOVA.

BMI = body mass index; DBP = diastolic blood pressure; HR = heart rate; SBP = systolic blood pressure; 24 h UK = 24 h urinary potassium; 24 h UNa = 24 h urinary sodium.

* The data did not fit a normal distribution. The differences between groups were assessed using the Kruskal–Wallis equality-of-populations rank test.

### 3.3. Association between genotype and BP response to saline loading

**Figure 1.**
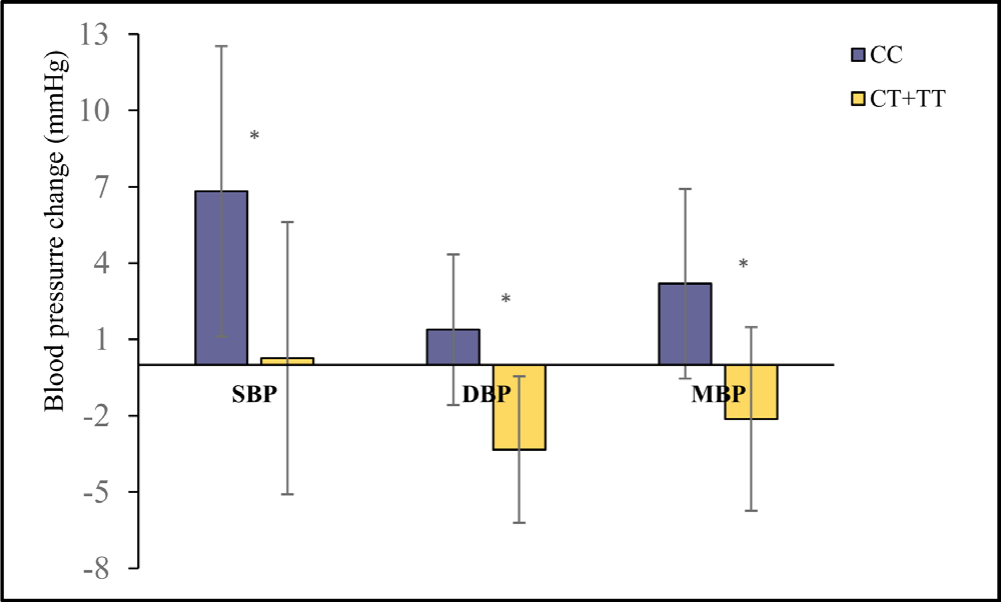
Adjusted BP response^‡^ by linear multivariable regression analysis. *Note*: Continuous variables were described as mean ± standard deviation (SD); difference in group change were record as mean, 95% CI; the results were adjusted for gender, age, smoking, drinking, and baseline BP. * *P*-value < .05. BP response^‡^ referred to the blood pressure value change between baseline and acute saline loading ending prolong for 3 hours (mean value for 4 timepoint, totally 12 times). BP = blood pressure.

All patients had their sitting BP measured 3 times at baseline to calculate the mean value. Immediately after NS loading ended, and then every hour for the next 3 hours, BP was measured 3 times. The BP response to NS loading was calculated as the mean of these 12 measurements minus the baseline value. The unadjusted BP response between 2 groups was shown in Table 4. There was no significant difference in baseline BP and HR across genotypes. However, individuals carrying the T allele demonstrated a more substantial tolerance to BP increase following 2 L saline infusion. Specifically, the SBP change for T allele carriers was significantly lower (0.26 ± 9.72 mm Hg) compared to CC genotype carriers (6.82 ± 11.65 mm Hg; *P* = .005). Similarly, DBP and mean arterial pressure (MAP) changes in T allele carriers were −3.35 ± 7.52 mm Hg and −2.13 ± 7.85 mm Hg, respectively, compared to 1.38 ± 7.62 mm Hg and 3.19 ± 7.81 mm Hg in CC genotype carriers (*P* = .003 and *P* = .001, respectively). These differences persisted after adjusting for gender, age, smoking, drinking, and baseline BP. The SBP increasing by 6.56 (4.30, 8.82) mm Hg, DBP by 4.70 (3.52, 5.88) mm Hg, and MAP by 5.32 (3.84, 6.81) mm Hg in the CC group, compared to the T carriers, as presented in Figure 1. The HR changes before and after NS loading remained nonsignificant between the 2 groups.

**Table 4:** Unadjusted BP response^‡^ according to acute saline loading between 2 groups.

Variable (c.214T > C)	CC (n = 129)	CT + TT (n = 29 + 1)	*P*
Baseline			
SBP	136.78 ± 512.12	140.47 ± 511.83	.324
DBP	88.94 ± 59.27	91.83 ± 58.98	.305
MAP	104.89 ± 9.10	108.04 ± 8.87	.232
HR	77.21 ± 10.62	75.94 ± 10.81	.841
BP response^‡^ according to acute saline loading			
△SBP	6.82 ± 11.65	0.26 ± 9.72	.014*
△DBP	1.38 ± 7.62	-3.35 ± 7.52	.011*
△MAP	3.19 ± 7.81	-2.13 ± 7.85	.005*
△HR	0.90 ± 9.37	0.24 ± 7.07	.936

*Note:* Continuous variables were described as mean ± standard deviation (SD), compared by one-way ANOVA analysis; BP response^‡^ referred to the blood pressure value change between baseline and acute saline loading ending prolong for 3 hours (mean value for 4 timepoints, totally 12 times).

BP = blood pressure; DBP = diastolic blood pressure; HR = heart rate; MAP = mean arterial pressure, MAP = (2 × DBP + SBP)/3; SBP = systolic blood pressure.

* *P*-value < .05.

### 3.4. Association between genotype and BP salt sensitivity

Salt sensitivity is defined as an increase in mean arterial pressure of 10 mm Hg or more following salt loading, or a decrease of 5 mm Hg or less after diuresis.^[20]^ The detection rate of salt sensitivity was 63.5%, with rates of 66.7% in the CC group and 50.0% in T allele carriers (*P* = .001). It appeared that individuals in the CC group were more likely to be salt-sensitive. These results were presented in Table 5.

**Table 5:** The salt sensitivity distribution in 2 groups.

Variable (c.214T > C)	CC (n = 129)	CT + TT (n = 29 + 1)	Total
Salt-sensitive	86 (66.7%)	15 (50.0%)	101 (63.5%)
Salt-no-sensitive	43 (33.3%)	15 (50.0%)	58 (36.5%)

### 3.5. Association between genotype and maximum BP after saline loading

**Figure 2.**
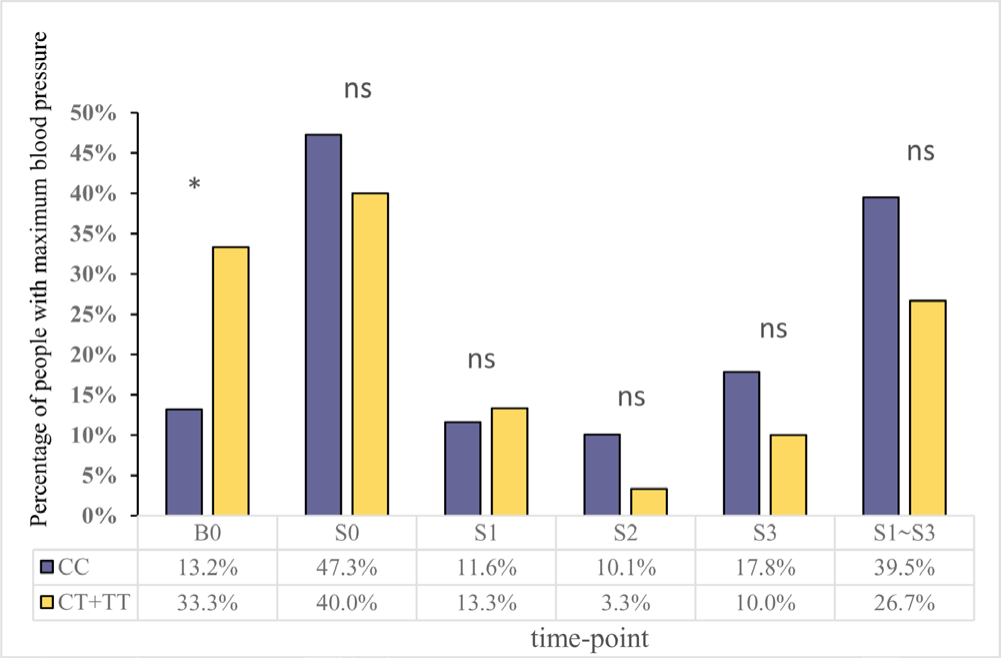
The distribution of different timepoints to maximum BP. *Note*: B0: baseline timepoint; S0: immediately at the end of normal saline loading; S1: 1 hour post-loading; S2: 2 hours post-loading; S3: 3 hours post-loading. * *P*-value < .05, suggesting statistical significance (Pearson Chi-square or Fisher exact test); “ns” denotes no significant difference (Pearson Chi-square or Fisher exact test). BP = blood pressure.

BP was measured at 5 distinct time points for each patient. The maximum BP was defined as the highest reading across these measurements, recorded at the corresponding time point. Detailed data on the time points of maximum BP for both groups are presented in Figure 2. Not all subjects experienced increased BP after NS loading: 112 (86.82%) in the CC group and 20 (66.67%) in T allele carriers did (*P* = .014). Among the 132 individuals, the time to reach maximum BP varied: at NS loading end, 61 (54.46%) in the CC group and 12 (40%) in T carriers; within the first hour post-loading, 15 (13.39%) in CC and 4 (20%) in T carriers; and so forth. There was a trend where T allele carriers (26.7%) were less likely to experience BP changes with 2 L NS loading, compared to CC genotype patients (39.5%), who were more likely to suffer delayed BP increases due to sodium retention, though this difference was not statistically significant.

## 4. Discussion

As ROS, superoxide anions, and NO rapidly interact to form peroxynitrite, which reduces NO bioactivity.^[21]^ Studies have shown that NO regulates sodium retention by inhibiting reabsorption in the kidney’s proximal tubule and thick ascending limb of Henle loop, mitigating salt-sensitive hypertension.^[8]^ Functional assays have demonstrated that the p22PHOX gene c.214T > C variant decreases the activity of the NOX, thereby reducing the production of ROS.^[13]^ Reduced oxidative stress from this genetic variant may decrease the risk of cardiovascular disease and salt-sensitive hypertension by maintaining higher NO bioavailability. However, population studies reveal conflicting results regarding the physiological impacts of this polymorphism. For instance, some studies^[22–24]^ suggested that the T allele reduce the risk of coronary artery disease. In contrast, Murate et al^[25]^ reported that T allele carriers have a higher odds ratio for atherothrombotic infarction, lacunar infarction, and transient ischemic attack among Japanese cardiovascular patients. Meanwhile, Cai et al^[26]^ found no association between this variant and coronary artery disease severity or history in Australian Caucasians.

Few studies have examined the BP response to acute NS infusion and its association with the p22PHOX gene c.214T > C variant. This study’s findings demonstrated that patients carrying the T allele exhibited increased tolerance to BP elevation after 2 L NS loading, with statistically significant differences in SBP, DBP, and MAP compared to those with the CC genotype. Analysis of the timing to maximum BP revealed that T allele carriers were less likely to experience saline-induced BP increases, whereas CC genotype patients more frequently showed delayed BP increases, typically occurring 2 to 3 hours post-infusion. Additionally, there were no significant differences in demographic characteristics between the 2 groups, particularly regarding variables linked to cardiovascular risk. Overall, the results of this study suggest that the p22PHOX T allele may serve as a protective factor against BP increases under acute saline loading. Conversely, several studies^[27,28]^ reported that the T allele is linked to increased salt sensitivity. These controversial findings likely stem from varied study contexts and methodologies. This study showed that the T allele is less prevalent in Chinese populations (frequency 0.0904), aligning with general East Asian trends, yet differs markedly from frequencies observed in European, African, and American populations (frequency ranges from 20–50%) according to publicly available databases.

This study suggests that hypertensive patients with the CC genotype are more susceptible to salt sensitivity. A modified acute salt load test,^[29,30]^ the detection of salt sensitivity in which was consistent with that in chronic saline loading test,^[31]^ was employed in this study. The current study could provide evidence for instantaneous BP changes during acute NS loading, which may influence the choice of infusion solvents and a patient’s capacity for increased cardiac output.

This study, while shedding light on the association between the p22PHOX gene variant and BP response to acute saline infusion, has limitations that warrant consideration. Notably, we did not measure urinary NO metabolite excretion, which could provide insights into the mechanism by which superoxide anion influences NO bioavailability.^[32]^ Second, renal blood flow theory, plasma renin levels, plasma aldosterone levels, and plasma angiotensin II concentrations were not included in this study. Future research should explore these information, alongside more comprehensive studies employing dietary salt protocols to better understand the relationship between genetic variants and salt sensitivity in hypertension.

## 5. Conclusion

This investigation into the relationship between the rs4673 (c.214T > C) and BP response to acute saline infusion provides valuable insights into the genetic underpinnings of salt sensitivity and hypertension. The study reveals that individuals carrying the T allele exhibit a significantly reduced BP increase following acute saline loading compared to those with the CC genotype. These findings suggest the T allele may offer a protective effect against the rise in BP induced by salt intake. This research underscores the importance of personalized medicine in the management of hypertension and the potential for genetic markers to guide dietary recommendations and treatment strategies.

## Acknowledgments

We extend our heartfelt gratitude to the medical professionals and researchers who contributed to this study, including Guoxi Wei and Hehui Hao from Gongyi City People’s Hospital, Liang An from Zhengzhou Yihe Hospital, Wenli Chen from Beijing Anzhen Hospital, and Nengjun Zhao from The Affiliated People’s Hospital of Inner Mongolia Medical University. Their dedication and expertise were instrumental in the completion of this research.

## Author contributions

**Data curation:** Hongyi Wang, Xue Wang, Yan Tian, Zhuo Wang.

**Methodology:** Hongyi Wang, Yan Tian, Zhuo Wang, Xiaoyan Nie, Ningling Sun.

**Project administration:** Xiaoyan Nie, Ningling Sun.

**Resources:** Li Yang, Xiaoxia Han, Ningling Sun.

**Software:** Xue Wang.

**Supervision:** Xiaoyan Nie, Ningling Sun.

**Writing – original draft:** Xue Wang.

**Writing – review & editing:** Hongyi Wang, Yan Tian, Li Yang, Xiaoxia Han, Zhuo Wang, Xiaoyan Nie, Ningling Sun.
